# The dissemination of motivational interviewing in Swedish county councils: Results of a randomized controlled trial

**DOI:** 10.1371/journal.pone.0181715

**Published:** 2017-07-27

**Authors:** Maria Beckman, Lars Forsberg, Helena Lindqvist, Margarita Diez, Johanna Enö Persson, Ata Ghaderi

**Affiliations:** 1 Department of Clinical Neuroscience, Division of Psychology, Karolinska Institutet, Stockholm, Sweden; 2 MIC Lab AB, Stockholm, Sweden; 3 Center for Psychiatry Research and Education, Karolinska Institutet and Stockholm County Council, Stockholm, Sweden; Brown University School of Public Health, UNITED STATES

## Abstract

**Objective:**

A significant number of Swedish practitioners are offered workshop trainings in motivational interviewing through community-based implementation programs. The objective of this randomized controlled trial was to evaluate to what extent the practitioners acquire and retain skills from additional supervision consisting of feedback based on monitoring of practice.

**Materials and methods:**

A total of 174 practitioners in five county councils across Sweden were randomized to one of the study's two groups: 1) Regular county council workshop training, 2) Regular county council workshop training followed by six sessions of supervision. The participant’s mean age was 43.3 years, and the majority were females (88.1%).

**Results:**

Recruiting participants proved difficult, which may have led to a biased sample of practitioners highly motivated to learn the method. Although slightly different in form and content, all the workshop trainings increased the participants’ skills to the same level. Also, consistent with previous research, the additional supervision group showed larger gains in proficiency compared to the group who received workshop training only at the six-month follow-up. However, analyses showed generally maintained levels of skills for all the participants at the follow-up assessment, and the majority of participants did not attain beginning proficiency levels at either post-workshop or follow-up.

**Conclusions:**

The results of this study address the real-life implications of dissemination of evidence-based practices. The maintained level of elevated skills for all participants is a promising finding. However, the low interest for obtaining additional supervision among the Swedish practitioners is problematic. In addition, neither the workshop trainings nor the additional supervision, although improving skills, were sufficient for most of the participants to reach beginning proficiency levels. This raises questions regarding the most efficient form of training to attain and sustain adequate practice standards, and how to create incentive and interest among practitioners to participate in such training.

## Introduction

The documented gap between research and practice has prompted increased efforts to disseminate and implement evidence-based practices (EBP) into community-based settings [1]. There is a wide range of different dissemination strategies in the literature [2], and it has been proved that successful implementation depends on multiple, interactive factors, both at the individual and organizational level [3]. Training programs for practitioners are one of the variables that affect the process of adopting EBP into routine care, but the training programs most frequently used do not always result in long-term changes in clinical practice [4, 5]. Motivational interviewing (MI), a collaborative, goal-oriented style of communication with particular attention to the language of change [6], is an EBP, widely used with a number of different clinical populations [7]. Meta-analyses of MI have reported small to medium effects on average, with significant efficacy in relation to substance use, smoking, short-term weight loss, gambling, and certain improved medical outcomes [8].

In Sweden, significant resources have been invested in implementing MI into health care, social services and correctional treatment. MI is also a key method in The Swedish National Board of Health and Welfare’s National Guidelines for Methods of Preventing Disease (socialstyrelsen.se/nationalguidelines). At the time of the study, the Swedish National Board of Health and Welfare was subsidizing MI trainings for employees in the Swedish county councils, 20 self-governed local authorities each corresponding to a county with responsibility for the public health care system and public transportation in its area. The resulting MI trainings varied slightly in form and content in the different county councils, gathering participants of diverse professions from a range of organizations with different social and organizational structures. The MI trainings extended from two to five days. Some of them included recording and transcribing of sessions. All had elements of exercises including role-plays. Some also included workshop enhancements where the participants brought recordings of practice samples to review together with the other participants in groups. However, evidence suggests that these traditional training approaches are relatively ineffective for integrating MI into clinical practice, and that training with additional supervision including systematic feedback using objective measures is more likely to sustain long-term proficiency in MI [7, 9–12]. Despite an increasing number of studies that focus on MI training, there is still a need for more research evaluating training offered through community-based implementation programs to practitioners from diverse professions.

The aim of this randomized controlled trial was to evaluate to what extent the practitioners in five Swedish county councils acquired and retained MI skills from regular workshop trainings, as opposed to workshop trainings followed by supervision consisting of feedback based on monitoring of practice. We hypothesized that the county councils workshop trainings would increase the participants’ skills in MI, and that the additional telephone supervision would contribute to a better long-term outcome than the workshop trainings alone.

## Materials and methods

Ethics approval was not required for this study since it does not include sensitive personal data as specified in chapter 3 of the Swedish Ethical Review Act (The Regional Ethical Review Board in Stockholm, Sweden, February 7, 2013; 2012/2195-31/5). The translated application for ethics approval and a supporting CONSORT checklist are available as supporting information: S1 File. Translated application for ethics approval, and S1 Table. CONSORT checklist. The authors confirm that all ongoing and related trials for this drug/intervention are registered. ClinicalTrials.gov registration number: NCT01197027. The study record was initially released February 2, 2014. Due to a miscommunication in the previous research group to which the first author belonged, the study did not receive a NCT number and was not finally released on the Clinical Trials public site until August 21, 2016. Written informed consent were obtained from all participants before enrolled in the study.

### Participants

**Fig 1:**
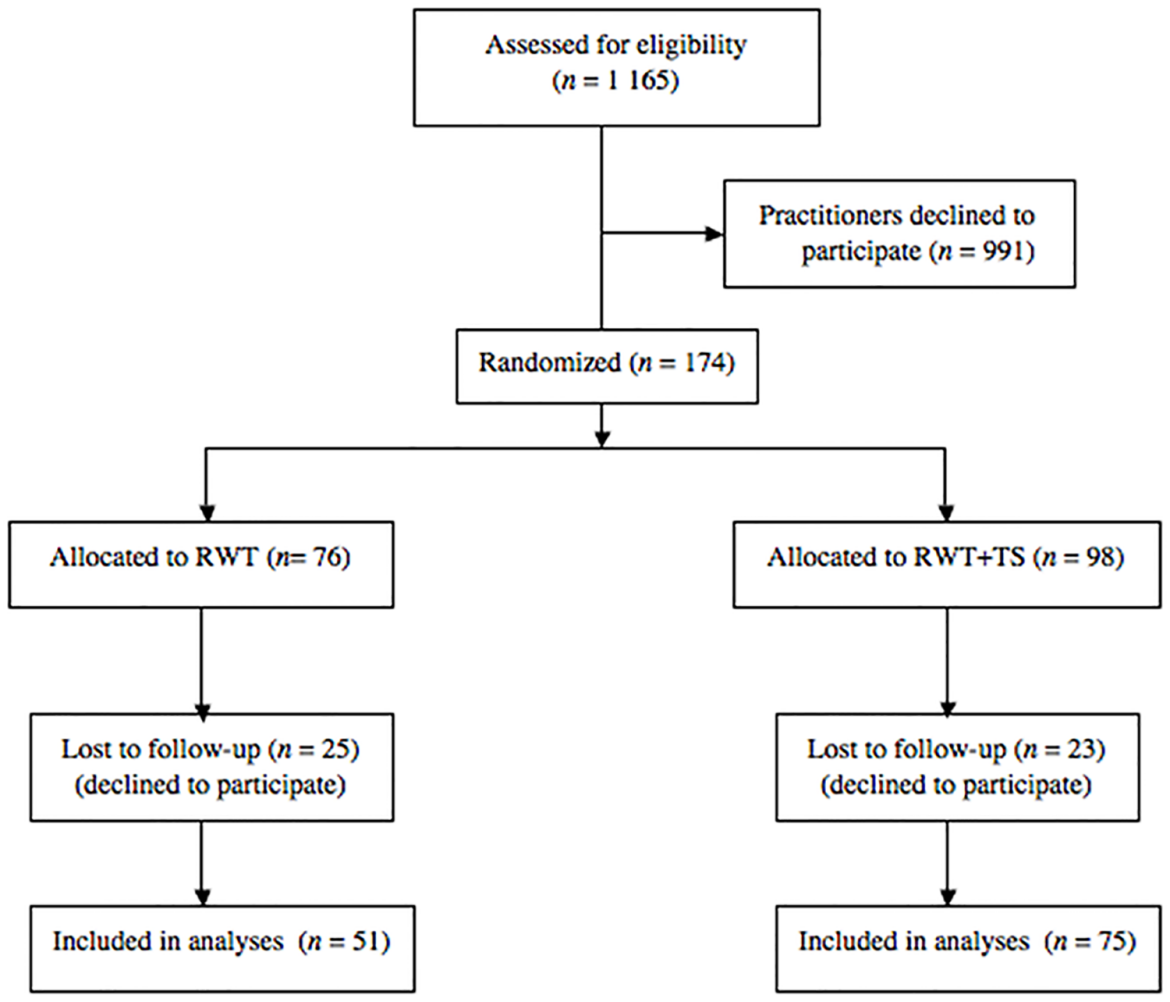
Flow chart of the participants in the study. RWT = regular county council workshop training, RWT+TS = regular county council workshop training.

The participants who completed the study (Fig 1) were 126 practitioners that attended MI training in five Swedish county councils during the date range for the study recruitment: January 1, 2013 to March 31, 2014. The end date for the study recordings (follow-up) and subsequent supervision was October 14, 2014. The mean age of the participants was 43.3 years (*SD* = 13.6), and the majority were females (*n* = 111, 88.1%). The education level varied from bachelor’s degree (*n* = 78, 61.9%) to master’s degree (*n* = 45, 35.7%). Two participants chose not to disclose their education level. The participants had a variety of occupations: nurses (*n* = 34), clinicians in social services (*n* = 18), physiotherapists (*n* = 18), teachers (*n* = 11), medical doctors (*n* = 10), counselors (*n* = 7), psychologists (*n* = 6), dietitians (*n* = 6), occupational therapists (*n* = 4), assistant nurses (*n* = 3), audiologists (*n* = 2), career counselor (*n* = 1), coach (*n* = 1), dental hygienist (*n* = 1), interpreter (*n* = 1), podiatrist (*n* = 1), and speech-language pathologist (*n* = 1). One participant chose not to disclose a profession.

### Procedure

All 1 165 practitioners who participated in the five county councils MI trainings during the study’s recruitment period were invited to the study by phone and email. The 991 practitioners, who due to various reasons chose not to participate, either declined or did not respond to at least three e-mails and one recruitment phone call. The 174 (14.9%) practitioners enrolled provided written informed consent and were randomized to one of the study's two groups (Table 1): 1) Regular county council workshop training (RWT) or 2) Regular county council workshop training followed by six additional individual monthly sessions of telephone supervision (RWT+TS). The randomization procedure was conducted across all the participants using a random number generator (1:1) without stratification. The researcher in charge of the randomization had no information or knowledge about the individual participants, and therefore no incentive to or possibility of affecting the randomization procedure. All the participants in the study recorded three 20-minutes sessions over phone with one of five actors role-playing standardized patients: One before the county councils’ workshop trainings (pre-workshop), one directly after the workshop trainings (post-workshop), and one 6 months after the workshop trainings (follow-up). The participants randomized to the RWT+TS group recorded five additional sessions with the actors between the post-workshop and the follow-up recording. Two days ahead of each recording, the participants received an email with information about the patient that would be role-played, and an outline of the session target behavior. The instruction they received before the pre-workshop recording was: “This is a baseline recording for an MI training trial. We therefore do not expect you to have any skills in MI. Just try to do your best /what you usually do”. The instruction they received before all the other recordings was: “Use the MI skills you’ve learned”. The rate at which the recordings and supervision was provided to the two groups is described in Table 2. The standardized patients, briefly described to the actors in scripts, were based on the lifestyle habits described in The Swedish National Guidelines for Methods of Preventing Disease (socialstyrelsen.se/nationalguidelines): tobacco use (session one and five), hazardous use of alcohol (session two and six), insufficient physical activity (session three and seven), and unhealthy eating habits (session four and eight).

**Table 1:** The practitioners invited and randomized to the two groups in the study.

Swedish county council (CC)	*n* invited to participate	*n* (%) agreed to participate	*n* dropouts (%)	RWT (%)	RWT+TS (%)
**CC1**	49	25 (51.0)	7 (28.0)	8 (44.4)	10 (55.6)
**CC2**	192	33 (17.2)	8 (24.2)	8 (32.0)	17 (68.0)
**CC3**	187	25 (13.4)	8 (32.0)	9 (52.9)	8 (47.1)
**CC4**	256	46 (18.0)	11 (23.9)	15 (42.9)	20 (57.1)
**CC5**	481	45 (9.4)	14 (31.1)	11 (35.5)	20 (64.5)
**Total**	1 165	174 (14.9)	48 (27.6)	51 (40.5)	75 (59.5)

*Note*. RWT = regular county council workshop training, RWT+TS = regular county council workshop training followed by six sessions of individual telephone supervision.

**Table 2:** Description of the study.

Assessment points									
	Pre-WT		Post-WT						6 moths follow-up
**RWT**	MITI	WT	MITI						MITI
**RWT+TS**	MITI	WT	MITI/ TS	MITI/ TS	MITI/TS	MITI/ TS	MITI/ TS	MITI/ TS	MITI
**SP scenarios**	Tobacco		Alcohol	Physical activity	Eating habits	Tobacco	Alcohol	Physical activity	Eating habits
***n* (%) TS completers**			84 (85.7)	82 (83.7)	77 (78.6)	76 (77.6)	75 (76.5)	72 (73.5)	

*Note*. RWT = regular county council workshop training, RWT+TS = regular county council workshop training followed by six monthly sessions of individual telephone supervision, MITI = motivational interviewing treatment integrity code, WT = the county councils workshop trainings, TS = telephone supervision, SP = standard patient.

### County councils’ workshop training

Five Swedish county councils, stretching from north to south, participated in the study. Participation was voluntary and without compensation. The five county councils had responded positively to an initiative funded by the National Board of Health and Welfare to evaluate the MI-training programs in the Swedish county councils. All the 20 Swedish county councils were approached, but 15 declined to have their programs evaluated due to time restraints or organizational difficulties. Throughout the study’s recruitment period, each county council’s training provider served the research group with lists of workshop participants. Using an online form, the training provider also described the form and content of the workshops in each county council (Table 3).

**Table 3:** Form and content of the county councils workshop trainings.

CC	Length of WT (days ^a^)	Didactic presenta-tions	Experien-tial exercises	Role-plays	WT enhance-ments	Tran-scripts	Video demo	MINT member
**CC1**	2 + 1 ^b^	Yes	Yes	Yes	Yes	Yes	Yes	No
**CC2**	2 + 1 + 1/2 ^c^	Yes	Yes	Yes	Yes	Yes	Yes	Yes
**CC3**	1 + 1 ^d^	Yes	Yes	Yes	Yes	No	No	No
**CC4**	2 + 1 ^e^	Yes	Yes	Yes	Yes	Yes	No	Yes
**CC5**	1 + 1 ^f^	Yes	Yes	Yes	Yes	Yes	No	Yes

*Note*. CC = Swedish county council, WT = workshop training, WT enhancements = an opportunity to bring recordings of a practice sample to review in smaller groups, Transcripts = transcriptions of the recorded practice samples, MINT = motivational interviewing network of trainers.

^a^ A standard 8-hour workday.

^b^ A month and a half apart.

^c^ One + three months apart.

^d^ Two and a half weeks apart.

^e^ One month apart.

^f^ Two weeks apart.

### Telephone supervision

A total of 12 coders at The Motivational Interviewing Coding (MIC) Lab at Karolinska Institutet, Sweden, worked with the monthly codings and 30 minutes sessions of telephone supervision. The participants were randomly assigned to a supervisor for each session (i.e. each supervisee had between 2 and 4 different supervisors across the study). For each session, the same supervisor both coded the recordings according to the motivational interviewing treatment integrity (MITI) code [13], and performed the supervision. The participants received the MITI protocol and were asked to listen to the recordings ahead of the supervision session. All the sessions were based on the results of the MITI, conducted in a manner consistent with MI and structured by a manual: The sessions started with an introduction and a collaboratively agreed upon agenda. The supervisor also reviewed homework from previous sessions during this initial segment. Then, referring to the results of the MITI, the supervisor provided performance feedback and initiated a discussion about consistent and inconsistent MI behaviors and gave opportunities for participant’s self-evaluation and input. The greatest focus was on the practice phase, where the supervisors used individualized role-plays based on segments from the recorded sessions and demonstrated specific skills to promote learning through observation. The supervisors then encouraged the participants to practice one or two specific skills as homework. At the end of each session, participants were asked to summarize and evaluate the session. Supervisors had supervisory meetings once a month throughout the study period to which they brought a self-selected recording of a supervision session.

### Assessment

The participant’s characteristics (i.e., gender, age, education level and profession) were assessed online with a self-reported questionnaire administered after obtaining an informed consent and the pre-workshop recordings. All the recorded 20 minutes sessions were assessed for proficiency in MI using the Swedish version of the MITI, version 3.1 [14].

The MITI is a coding system with good psychometric properties [13, 15], widely used as a treatment integrity measure and as a feedback tool to improve MI skills in training and in clinical practice. MITI 3.1 consists of two main components: (1) The five global dimensions (Empathy, Evocation, Collaboration, Autonomy and Direction) that reflects the coder’s overall judgment of the practitioner’s performance on a 5-point scale, and (2) The behavior counts, which are frequency counts of every utterance by the practitioner coded in seven specified categories (Giving information, MI adherent behaviors, MI non-adherent behaviors, Closed questions, Open questions, Simple reflections and Complex reflections). The behavior counts affect the global scores, but there is no direct correlation between them. Table 4 shows the recommended indicators of MITI beginning proficiency and competency to aid the evaluation of clinicians’ skillfulness in MI [16]. The coders were not blind to the participants’ group allocation. Throughout the study, all the coders at MIC Lab received 120 hours of initial stepped training and participated in group-coding sessions each week throughout the study period to reach and maintain MITI inter-rater reliability. Also, as a part of regular MIC Lab coding practice, 12 randomly selected recordings sent to the lab are twice a year double-coded by all the coders at MIC Lab to assess and ensure high inter-rater agreement according to Cicchetti’s [17] system for evaluating intraclass correlations (ICC). At the middle of the study period, in January 2014, the MIC Lab ICCs ranged between good to excellent for all the MITI variables except for Direction and MI non-adherent, for which ICC was considered fair (Table 5).

**Table 4:** Recommended proficiency and competency thresholds for clinicians according to MITI 3.1.1.

MITI summary scores	Beginning proficiency	Competency
**Global clinician ratings**	Average of 3.5	Average of 4
**Reflection to question ratio (R:Q)**	1	2
**Percent open questions (%OC)**	50%	70%
**Percent complex reflections (%CR)**	40%	50%
**Percent MI-adherent (% MIA)**	90%	100%

*Note*. MITI = motivational interviewing treatment integrity code.

**Table 5:** Inter-rater reliability for the MITI 3.1 variables at the middle of the study period, January 2014.

The MITI 3.1 variables	ICC
**Empathy**	.60
**Evocation**	.69
**Collaboration**	.74
**Autonomy**	.75
**Direction**	.49
**Giving information**	.89
**MI adherent behaviors**	.81
**MI non-adherent behaviors**	.59
**Closed questions**	.64
**Open questions**	.92
**Simple reflections**	.73
**Complex reflections**	.68

*Note*. MITI = motivational interviewing treatment integrity code, ICC = intra-class correlation coefficient. According to Cicchetti [17], an ICC below .40 is considered poor, between .40–.59 fair, between .60–.74 good, and between .75–1.00 excellent.

### Data analysis

The primary outcomes for this trial were the summary values for each of the groups on each of the seven MITI proficiency measures at pre-training, post-training, and at the six-month follow-up assessment. The power analysis showed that we needed to include 200 participants to attain a power of at least .80 with p < .05, and a between-group effect size of .40. All data were analyzed using the Statistical Package for the Social Sciences (SPSS), version 22.0. Descriptive statistics were generated and presented as frequency, mean (SE) or percent. The inter-rater agreement of the MITI coding was estimated by calculating ICCs, inserting single measures and employing the two-way mixed model with absolute agreement. All outcomes based on MITI were examined independently for main effects (i.e., group, and time), and for interaction (group X time). Participants with missing data were only excluded from the presentation of demographics. As participants completed the demographic questionnaire after the first recording, and most dropouts occurred after the first recording, we unfortunately only got a few of the dropouts to fill out the forms. To test the relationship between the participants’ level of adherence to the original study schedule and their MI skills acquisition, two new variables was created (level of adherence and MI skills acquisition). Spearman's rank correlation was then used to test the relationship between the participant’s level of adherence and their MI skills acquisition at the post-workshop assessment and at the 6 months follow-up. To test the effectiveness of the county council workshop trainings from pre- to post-workshop, and the impact of the additional supervision from post-workshop to follow-up, a generalized linear mixed model (GLMM) was conducted to control for the nested nature of the data (i.e., to customize the covariance structure so it would reflect the nature of repeated measures) and to efficiently handle missing data. Using QQ-plots and other descriptive statistics, the distribution that most accurately represented data was chosen. The patterns of missing data were also examined to reach a decision on strategy for handling missing data at item level. We used the identity link for the normal covariance structure and log link for the gamma distribution. Beyond nesting, which is done for repeated measures, and random intercept for individuals, other adaptations such as random slope or nesting within the councils did not provide a better fit for data. Since the growth curve usually is different for a short, intensive period of learning (e.g., pre- to post-treatment), compared to a longer period with stabilization and maintenance of skills in focus (e.g., during the follow-up period), in line with running piecewise-regression models in such circumstances [18], the outcome was investigated using two models: one from pre- to post-workshop, and another from post-workshop to follow-up. All primary outcomes were examined for main effects (i.e., group, and time), and for interactions (group X time). The Bonferroni correction was applied for multiple comparisons and the magnitude of the intervention effect was determined using Cohen’s d effect size estimates. Chi-square analyses were also employed to examine the differences of the proportion of clinicians who met the MITI beginning proficiency thresholds in the study’s two groups.

## Results

### Participant attrition and compliance

Only 174 (14.9%) of the 1 165 invited practitioners agreed to participate in the study, of which 126 (72.4%) completed all of their recorded sessions (Table 1). The attrition rate was higher in the RWT group (32.9%) than in the RWT+TS group (23.5%). Twelve (25,0%) of the 48 participants who chose not to complete their participation stated scarcity of time as the reason, one stated not liking the method (MI), and 35 (72.9%) were inaccessible, despite repeated attempts to reach them by both telephone and email. Of the 48 participants who dropped out, 26 (54.2%) recorded only one of the sessions, 15 (31.3%) recorded two, four (8.3%) recorded three, and three (6.3%) recorded four sessions. Demographic comparisons could not be performed between those who did and did not complete the study because only six (12.5%) of the 48 responded to and submitted their questionnaires. However, independent t-tests were conducted to compare the pre-workshop assessment of proficiency in MI between those who did and did not complete the study. Mean differences between the two groups were small (ranging from 0.01 to 0.13) and non-significant across the seven MITI summary scores. Moreover, due to various reasons, mostly stated as lack of time, some participants could not always conduct the monthly recordings and following supervision according to the original study schedule (Table 2). As a result, certain time lags occurred. Some participants (n = 26, 34.7%) even recorded the following session before they received supervision for the prior. For 12 of these 26 participants, this occurred two or more times. However, we found no correlations between the participants’ level of adherence to the original study schedule and their MI skills acquisition, neither at the post-workshop assessment nor at the 6 months follow-up, which suggests that these time lags did not exert a significant influence on outcome. Despite these time lags, the follow-up sessions were recorded 6 months (mean = 27.71 weeks, *SD* = 2.29) after the last day of the workshop trainings.

### Effectiveness of the county council workshop trainings

All the five county councils workshop trainings increased the participants’ skills in MI with higher means for all the seven MITI proficiency measures except for MI adherent behaviors and Percent complex reflections at the post-workshop assessment. Mean differences between the pre- and post-workshop assessment ranged from −0.06 to 3.49 across the proficiency measures, and the GLMM-analysis showed significant time effects for five of the seven MITI proficiency measures: Empathy (F (1, 309) = 67.31, *p* < .001, *d* = 1.46), MI spirit (F (1, 309) = 78.28, *p* < .001, *d* = 1.58), MI non-adherent behaviors (F (1, 309) = 119.26, *p* < .001, *d* = 1.95), Reflection to question ratio (F (1, 309) = 42.96, *p* < .001, *d* = 1.19), and Percent open questions (F (1, 309) = 13.71, *p* < .001, *d* = 0.66). In addition, Bonferroni adjusted comparisons indicated some differences among the five county councils at the pre-workshop assessment, with significantly lower baseline skill levels for three of the proficiency measures (Empathy, MI spirit and MI non-adherent behaviors) for county council one (CC1) compared to all the other county councils. However, at the post-workshop assessment, the only significant difference was between county council two (CC2) and five (CC5) for only one of the proficiency measures (MI adherent behaviors). Moreover, the GLMM-analysis showed no significant group effects, but significant interaction effects between time (i.e., from pre- to post-workshop) and group (i.e., the county councils) for three of the seven MITI proficiency measures: MI spirit (F (4, 309) = 2.91, *p* = .02, *d* = .30), MI adherent behaviors (F (4, 309) = 3.03, *p* = .02, *d* = .31), and MI non-adherent behaviors (F (4, 309) = 6.56, *p* < .001, *d* = .46). Since the Bonferroni adjusted comparisons indicated only one significant difference (between CC2 and five CC5) for only one of the proficiency measures (MI adherent behaviors) at the post-workshop assessment, these significant interactions are explained by the exhibited differences at the pre-workshop assessment.

### Impact of additional telephone supervision on the county councils’ workshop trainings

At baseline, independent t-tests showed no differences between the two groups for neither the baseline characteristics (i.e., gender, age, education level and profession) nor any of the seven MITI proficiency measures (Table 6). The GLMM-analysis showed significant interactions effects between time (i.e., from post-workshop to follow-up) and group (i.e., RWT and RWT+TS) for three of the seven MITI proficiency measures: MI spirit (F (1, 266) = 7.12, *p* < .01, *d* = 0.48), Percent open questions (F (1, 266) = 5.06, *p* < .05, *d* = 0.40), and Percent complex reflections (F (1, 264) = 14.77, *p* < .001, *d* = 0.68). Post-hoc contrasts then showed significantly higher means for the RWT+TS group for six of the seven MITI proficiency measures at the follow-up assessment (Table 6). The GLMM-analysis also showed significant time effects for three of the seven MITI proficiency measures: MI adherent behaviors (F (1, 266) = 16.49, *p* < .001, *d* = 0.72), MI non-adherent behaviors (F (1, 266) = 4.37, *p* < .05, *d* = 0.37), and Percent open questions (F (1, 266) = 17.76, *p* < .001, *d* = 0.75), and significant group effects for four of the seven MITI proficiency measures: Empathy (F (1, 266) = 5.52, *p* < .05, *d* = 0.42), MI spirit (F (1, 266) = 7.23, *p* < .05, *d* = 0.48), MI non-adherent behaviors (F (1, 266) = 9.08, *p* < .05, *d* = 0.54), and Reflection to question ratio (F (1, 266) = 6.05, *p* < .05, *d* = 0.44).

**Table 6:** Group means of MITI proficiency measures for the two groups in the study.

MITI measures	Pre-training (SE)	Post-training (SE)	Follow-up (SE)
**Empathy**			
**RWT**	2.67 (0.10)	3.30 (0.10)	3.40 (0.11)^a^*
**RWT+TS**	2.71 (0.09)	3.52 (0.09)	3.70 (0.09)^a^*
**MI spirit**			
**RWT**	2.36 (0.09)	3.08 (0.10)	2.92 (0.10)^a^***
**RWT+TS**	2.53 (0.08)	3.18 (0.08)	3.40 (0.09)^a^***
**Adherent behaviors**			
**RWT**	3.67 (0.18)	3.31 (0.18)	3.79 (0.22)
**RWT+TS**	3.61 (0.15)	3.34 (0.15)	4.18 (0.20)
**Non-adherent behaviors**			
**RWT**	6.62 (0.48)	3.58 (0.29)^a^*	4.14 (0.36)^a^**
**RWT+TS**	6.11 (0.39)	2.89 (0.20)^a^*	3.12 (0.22)^a^**
**Reflection to question ratio**			
**RWT**	2.61 (0.06)	2.87 (0.07)	2.79 (0.08)^a^**
**RWT+TS**	2.57 (0.05)	3.03 (0.06)	3.06 (0.07)^a^**
**% Open questions**			
**RWT**	36.41 (1.54)	42.94 (2.35)	46.92 (2.52)^a^**
**RWT+TS**	37.28 (1.87)	43.13 (2.06)	56.07 (2.16)^a^**
**% Complex reflections**			
**RWT**	45.64 (1.76)	50.25 (2.53)^a^**	43.91 (2.87)^a^**
**RWT+TS**	45.50 (1.75)	42.03 (2.23)^a^**	52.44 (2.35)^a^**

*Note*. MITI = motivational interviewing treatment integrity code, MI = motivational interviewing, RWT = regular county council workshop training, RWT+TS = regular county council workshop training followed by six sessions of individual telephone supervision.

^a^ The two groups differs significantly at this time point on these MITI summary scores after Bonferroni correction. Significance levels:

*p < 0.05,

**p < 0.01,

***p < 0.001.

### Proficiency thresholds

Table 7 shows the number and proportion of participants reaching the MITI beginning proficiency thresholds in the two groups, at each assessment point. Some participants achieved beginning proficiency levels on single indicators at the pre-workshop measurement, with results varying widely between the thresholds from 8.2 and 9.4 percent for Percent MI-adherent, to 66.8 and 67.4 percent for Percent complex reflections. At the post-workshop assessment, the number of participants achieving beginning proficiency levels increased for both groups, for all thresholds except Percent complex reflections, where the proportion of participants in the RWT+TS group decreased from 67.4 to 55.4 percent. Still at this time point, Percent complex reflections showed the highest proportion of participants reaching beginning proficiency thresholds.

**Table 7:** The participants in the study’s two groups reaching the MITI beginning proficiency thresholds.

Measure	*n* (%) proficient pre-workshop	*n* (%) proficient post- workshop	*n* (%) proficient follow-up
**Global clinician ratings**			
**RWT**	14 (18.4)	29 (47.5)	20 (40.0)*
**RWT+TS**	18 (18.4)	44 (52.4)	46 (61.3)*
**Reflection to question ratio**			
**RWT**	13 (17.1)	22 (36.1)	15 (30.0)
**RWT+TS**	12 (12.2)	34 (40.5)	35 (46.7)
**Percent open questions**			
**RWT**	15 (19.7)	26 (42.6)	24 (48.0)*
**RWT+TS**	26 (26.5)	34 (40.5)	51 (68.0)*
**Percent complex reflections**			
**RWT**	50 (66.8)	47 (78.3)	25 (50.0)*
**RWT+TS**	66 (67.4)	46 (55.4)	58 (77.3)*
**Percent MI-adherent**			
**RWT**	6 (8.2)	19 (35.8)	13 (28.9)
**RWT+TS**	9 (9.4)	34 (51.5)	31 (47.0)

*Note*. MITI = motivational interviewing treatment integrity code, RWT = regular county council workshop training, RWT+TS = regular county council workshop training followed by six sessions of individual telephone supervision.

*The two groups differ significantly at the 0.05 level at this time point on these MITI proficiency measures.

At follow-up, the proportion of participants reaching beginning proficiency in the RWT group decreased for all thresholds except Percent open questions, while the proportion of participants reaching proficiency in the RWT+TS group increased for all thresholds except Percent MI-adherent. Also at this time point, Percent complex reflections showed the highest proportion of participants achieving proficiency thresholds, suggesting that this was the easiest threshold for the participants to reach.

## Discussion

This study aimed to assess to what extent the practitioners acquire and retain MI skills from the workshop trainings offered through community-based implementation programs in Swedish county councils, and from workshop trainings with additional supervision consisting of feedback based on monitoring of practice.

### Increased skills following the workshops

Regardless of their slightly different form and content and despite some differences in proficiency level at the pre-training assessment, all the five workshop trainings increased the participants’ skills in MI to virtually the same level. These findings are consistent with previous research demonstrating that workshop trainings can improve skills in MI [7, 11], and indicate that the results also apply to naturalistic settings. Compared with previous studies [19–25], the results also revealed a relatively high baseline skill level among all participants (Table 6). These high baseline skill levels might be explained by a sampling bias as only 14.9% of the practitioners approached chose to participate in the study. In addition, the county council with the lowest baseline skill levels (CC1) had the highest percentage (51%) of participating practitioners (Table 1). This indicates that the study participants might have been more interested and motivated to learn the method, and also more proficient, than the larger population of county council practitioners. Also noteworthy is the fact that, at the post-training assessment, CC1 reached the same levels of proficiency as the other county councils despite lower pre-training scores. Previous MI training studies have conversely suggested baseline skill levels as a possible predictor of post-workshop performances [25–28].

### Increased skills following the additional supervision

The additional telephone supervision contributed to a better long-term outcome than the county councils’ workshop trainings alone. The analyses showed significantly higher means for the RWT+TS group for six of the seven MITI proficiency measures at the follow-up assessment (Table 6). These findings are consistent with previous research showing that additional coaching and feedback following workshop training in MI can help to maintain and improve post-workshop proficiency [7, 11]. However, at the follow-up assessment, the analyses also showed generally maintained levels of skills for the RWT group (Table 6). These results may be due to the sample of self-selected participants, but are nonetheless surprising and contradict the general picture in current MI-training literature that trainees need additional coaching and feedback to retain proficiency in MI [7, 11]. However, some other previous studies have also found that not all MI training workshop participants need additional coaching and feedback to maintain [19, 23] or increase [29–31] their gained MI skills. Additionally, some previous studies have found that not all additional coaching and feedback efforts lead to retained or increased skill levels [25, 32, 33].

### Proficiency levels

Percent complex reflections showed the highest proportion of participants reaching proficiency thresholds across all the study’s assessment points, which again contradicts the general picture in the current MI-training literature [34], where complex reflections is a skill that has proved challenging for practitioners to learn. The high baseline skill levels and/or a sampling bias might explain also this finding. At follow-up, the RWT+TS group had a higher percentage of participants reaching beginning proficiency levels at all the proficiency thresholds. However, despite the high baseline skills and the increased post-workshop skills, the majority of participants did not attain beginning proficiency levels at either the post-workshop or the follow-up assessment. The majority of participants in the RWT+TS group attained proficiency at follow-up on three of the five thresholds, and a majority of the RWT group attained proficiency in one of the five, though that score did not represent an improvement from baseline (Table 7). Other MI training studies have similarly showed difficulties for participants to reach beginning proficiency levels [19–21, 23, 35]. In a recent systematic review [36], only two out of 20 studies met the criteria of 75% of clinicians achieving beginning proficiency in MI spirit after training. In both these studies, training and supervision continued until competency was met.

This study has several limitations. First, the sample of self-selected participants may not well represent the larger population of county council practitioners, which limits the generalizability of the findings. The recruitment efforts involved at least three e-mails and one phone call. A more intensive recruitment had become too costly, but may have led to greater involvement in the study. Second, when studying the effectiveness of the county council workshop trainings, the absence of a comparison group and the fact that the participants were not randomized to the five groups presents a number of threats to the study’s internal validity. Third, there was only one recording per assessment point. Since MI performance often varies significantly within therapists [37, 38], repeated measures at each assessment point would have assured a more accurate estimates of the participants’ MI integrity. Forth, using standardized patients does not provide adequate information about how MI is employed in actual clinical practice; Decker and colleagues [39] found that clinicians were significantly more MI adherent and used more advanced MI strategies in role-played sessions than in real client sessions, and demonstrated poor rating correspondence between the two assessment approaches. However, standardized patients allow for clients’ characteristics to be kept constant between both participants and assessment points, indicating that, if not used interchangeably with real client sessions, role-played sessions can provide useful information about therapists’ MI performance [38, 39]. Fifth, the follow-up assessment took place six months after the post-workshop assessment. A follow-up period longer than six month would have given a better picture of how participants' MI skills are sustained over time. Sixth, within the scope of this study, it was not possible to also evaluate the impact of organizational-level variables. However, only 14.9 percent of the practitioners agreed to participate, out of which 27.6 percent dropped out. The most common stated reason for not participating or completing the study was time constraints. Additionally, the participants who completed the study frequently reported time pressures and, in some instances, insufficient organizational support. These contextual challenges for learning transfer should be addressed in future dissemination studies. Seventh, the coders in this study were not blind to the participants’ group allocation during coding, and the same sample of coders rated sessions and performed supervision with the participants. This may have affected the reliability of the coding. However, the coders at MIC Lab are experienced in assessing and supervising the same practitioners as part of regular practice. Despite these limitations, the present study contributes to the knowledge of dissemination of MI by being one of few studies that evaluates MI training using a relatively large sample of practitioners in a naturalistic setting. It can therefore provide some direction and considerations for future MI dissemination studies.

## Conclusions

In accordance with previous research, the present study showed that workshop training can increase participants’ MI skills, and that workshop trainings including subsequent supervision can produce better outcome than workshop trainings alone. Additionally, it indicates that these results also apply to naturalistic settings. However, the high variation in competence at all the assessment points and the low interest in the possibility of additional supervision among the county council practitioners are troublesome. In addition, neither the workshop trainings, nor the costly additional six sessions of individual telephone supervision were sufficient for most of the participants to reach beginning proficiency levels. Although the levels of MI proficiency sufficient for making a difference in client outcomes is unclear [8], this raises questions regarding both the most efficient form of training for practitioners to attain and sustain adequate practice standards, and how to create an interest among practitioners to participate in such training. Martino and colleges’ [27] evaluation of a stepwise approach for MI training found that different participants required different form and types of training to learn adequate MI skills. Matching participants’ training needs to specific training strategies might be a way to manage both the high variation of skills and the low levels of participants reaching the beginning proficiency levels, and thus provide a more successful implementation of MI. A recurring theme in the recruitment of participants and in the collection of data was the participants' perceived scarcity of time for both the recordings and the supervision. Integrating a more flexible e-learning in both initial training and ongoing supervision could possible make the transfer of new skills into existing practice more accessible, helping the practitioners integrate the training into the daily workflow. Alternate training methods such as these, together with effective and affordable methods for assessing provider skills and ways to match type and amount of training to distinct participant training needs are all important aspects of future studies.

## Supporting information

S1 File Translated application for ethics approval. (PDF)

S1 Table CONSORT checklist. (DOC)

S2 Table Data set. (XLS)
