# Supplementary material for: The dissemination of motivational interviewing in Swedish county councils: Results of a randomized controlled trial
Source: PLoS One. 2017 Jul 27;12(7):e0181715. doi: 10.1371/journal.pone.0181715 (PMC5531530; doi:10.1371/journal.pone.0181715)
Supplement: S1 File — (PDF) [file pone.0181715.s001.pdf]

Translated application for ethics approval

To the Regional Ethical Review Board in: Stockholm, Sweden

Project title: *Implementation of Motivational Interviewing in Sweden; evaluation and development*

1. Information concerning the body principally responsible for the research etc.

1:1 The responsible research body: Center for Psychiatry Research and Education, Karolinska Institutet and Stockholm County Council, Liljeholmstorg 7B, 117 63 Stockholm, Sweden.

1:2 Qualified representative of the body principally responsible for the research: Jan Hillert, Head of department, Department of Clinical Neuroscience, Division of Psychology, Karolinska Institutet, 171 76 Stockholm, Sweden.

1:3 The researcher/s primarily responsible for conducting the project (principal contact person): Lars Forsberg, Center for Psychiatry Research and Education, Karolinska Institutet and Stockholm County Council, Liljeholmstorg 7B, 117 63 Stockholm, Sweden. Phone: + 46 (736) 923 050 lars.forsberg.3@ki.se

1:4 Arena: Motivational Interviewing Coding (MIC) lab, Center for Psychiatry Research and Education, Karolinska Institutet and Stockholm County Council, Liljeholmstorg 7B, 117 63 Stockholm, Sweden, and in five county councils in Sweden: Skåne, Stockholm, Sörmland, Värmland and Västernorrland.

1:5 Other collaborators: Only one principal contact person.

2. Information concerning the project

2:1 Summary of the research project: The basis for a successful implementation of an evidence-based treatment (EBT) in clinical care is that the underlying research on the method includes careful quality assessment of all stages of delivery, implementation and evaluation. Motivational Interviewing (MI) is an EBT with a firm evidence for a wide variety of clinical problems. In recent years, a vast amount of resources has been invested in implementing MI in health care, social services and the correctional treatment in Sweden. MI is also a key method in The National Board of Health and Welfare's recommendations for evidence-based methods of preventing disease in The National Guidelines for Methods of Preventing Disease.

In the Swedish county councils, MI training with slightly different form and content is taking place as part of the implementation of the method. The different training formats extend over two to five days, and some of them include recording and transcribing of sessions. All

trainings have elements of various forms of exercises including role-plays, but only some of them include supervision as part of the training. Evidence suggests that these traditional approaches to training are relatively ineffective for integrating MI into clinical practice, and that training that includes supervision based on monitoring of practice is more likely to enhance skills.

The overall objective of the proposed research is to systematically evaluate MI-training as part of the implementation of the method in five Swedish county councils to increase the knowledge of how cost-effective training in MI should be conducted in order to reduce unhealthy habits, improve healthy habits, and to improve compliance in the treatment of chronic diseases. This overarching objective has several different aspects:

1) To evaluate to what extent the practitioners acquire and retain MI skills from the different training methods used in the Swedish county councils by comparing them with a format that in previous studies has shown to be required for the long-term acquisition of proficiency in MI; training including supervision consisting of feedback based on monitoring of practice.

2) To examine if supervision including feedback based on only the behavior counts component of the Motivational Interviewing Treatment Integrity (MITI) Code, compared to supervision based on both components of the MITI, affect the participants experience of the supervision and is related to their ability to acquire and retain proficiency in MI. MITI is a coding system with good psychometric properties, widely used as a treatment integrity measure and as a feedback tool to improve MI skills in training and in clinical practice. MITI has two components: (a) The five global dimensions (empathy, evocation, collaboration, autonomy and direction) to capture the evaluators overall judgment of the practice sample on a scale ranging from 1 to 5, and (b) the behavior counts which are frequency counts of every utterances by the practitioner coded in seven different categories. Both global scores and behavior counts are assessed within a 20-minute segment of the audio-recorded interview.

3) To examine to what extent practitioner’s self-reports of proficiency in MI are consistent with objective assessment, and if the practitioners could be trained to more accurately assess their own performance by training in MI including supervision consisting of feedback based on monitoring of practice. Earlier research has found practitioners self-reports a poor indicator of actual MI adherence.

4) To examine to what extent pre-training levels of empathy predicts the practitioners’ capabilities to acquire and retain proficiency in MI, to serve as a part of a more effective individually tailored training in MI and as a potential selection variable in a future screening procedure for MI training.

5) To examine if the participants' levels of self-efficacy is related to the different training methods used in the Swedish county councils and to training including supervision consisting of feedback based on monitoring of practice.

6) To examine if the participants' perceived feeling of organizational support affect their ability to acquire and retain proficiency in MI

2:2 What is/are the primary scientific question(s) forming the basis of the design of the project? Hypothesis: Practitioners will acquire and retain MI skills from the different training methods used in the Swedish county councils; the practitioners randomized to additional supervision consisting of feedback based on monitoring of practice will show larger gains in proficiency compared to the group who receives workshop training only at the six-month follow-up assessment; supervision including feedback based on only the behavior counts component of the MITI will not affect the participants experience of supervision and will not affect the participants ability to acquire and retain proficiency in MI; practitioner's self-reports of proficiency in MI will not be consistent with objective assessment, but training and supervision in MI will make the practitioners more accurate in their own assessments over time; pre-training levels of empathy will predict the practitioners' capabilities to acquire and retain proficiency in MI – the practitioners with higher pre-training levels of empathy will show larger gains in proficiency compared to the practitioners with lower pre-training levels; the participants' level of self-efficacy will increase after MI training and MI training including additional supervision; participants' perception of training support from the organization they work in predicts their ability to acquire skills – the practitioners with support will show larger gains in proficiency after MI training and MI training including additional supervision.

2:3 State the results from relevant animal experiments (clinical trials): N/A

2:4 Give an overview of the examination procedures used, data collection and the nature of the data: Practitioners who participates in the five county councils MI trainings during the period of January first 2013 to June 31 2014 will be invited to the study by phone and email. The practitioners who agrees to participate will be randomized to one of the study's two groups: 1) *n* = 100, Regular county council workshop training (RWT) or 2) *n* = 100, Regular county council workshop training followed by six additional individual monthly sessions of telephone supervision (RWT+TS). All participants in the study will record three 20-minutes sessions over phone with one of five actors role-playing standardized patients: One before the county councils' workshop trainings (pre-workshop), one directly after the workshop trainings (post-workshop), and one 6 months after the workshop trainings (follow-up). The participants randomized to the RWT+TS group will record five additional sessions with the actors between the post-workshop and the follow-up recording. The 20 minutes session is followed by a short interview regarding self-reports of proficiency (Appendix 5b). Overall, the recording and the interview takes 30 minutes, after which the actor will upload the recording at MIC Labs website. Two days ahead of each recording, the participants will receive an email with information about the patient that would be role-played, and an outline of the session target behavior. The instruction to the practitioners before the pre-workshop recording: "Do what you usually do with your patients", and the instruction before all the other recordings: "Use the MI skills you've learned". The standardized patients will briefly be described to the actors in scripts, and will be based on the lifestyle habits described in The Swedish National Guidelines for Methods of Preventing Disease (socialstyrelsen.se/nationalguidelines): tobacco use (session one and five), hazardous use of alcohol (session two and six), insufficient physical activity (session three and seven), and unhealthy eating habits (session four and eight).

The coders at The Motivational Interviewing Coding (MIC) Lab at Karolinska Institutet, Sweden, will perform all the study's monthly codings and 30 minutes sessions of telephone supervision. The participants will be randomly assigned to a supervisor for each supervision session, and all the sessions will be based on the results of the MITI, conducted in a manner consistent with MI and structured by a manual. All the coders at MIC Lab have received a 120 hours of initial stepped training and participates in group-coding sessions each week to reach and maintain MITI inter-rater reliability. Also, as a part of regular MIC Lab coding practice, 12 randomly selected recordings sent to the lab are twice a year double-coded by all the coders at MIC Lab to assess and ensure high inter-rater agreement according to Cicchetti's system for evaluating intraclass correlations (ICC).

All the recorded 20 minutes sessions will be assessed for proficiency in MI using the Swedish version of the MITI, version 3.1. (Appendix 13). In conjunction with the initial county council trainings and the study's last recordings, all participants will sign an informed consent (Appendix 4) and complete a number of questionnaires regarding: a) Demographic variables (i.e., gender, age, education level and profession) (Appendix 5a), b) Form and content of the county council trainings including potential expanded exercises (Appendix 5a), c) Self-efficacy (Appendix 5c), and d) Organizational support (Appendix 5e). After each supervision session, participants will also answer questions regarding their emotional experience of the supervision (Appendix 14 and 15).

2:5 Describe how collected biological material is to be stored in a bio bank: N/A

2:6 Funding of the study: The Swedish National Board of Health and Welfare fund this study.

2:7 Documentation, data protection and record keeping: All names and personal data will be replaced with a code number in order to de-identify all participants. These code lists will be kept locked up in a filing cabinet at Karolinska Institutet. Lists of personal numbers, names, addresses, the informed consent and all questionnaires will also be kept locked in a filing cabinet at Karolinska Institutet. When uploaded at MIC Labs website, the recordings renames with the participant's code number. All MIC Lab web connections are encrypted. The de-identified data will be entered into a data file by the research team. The material that will be processed and analyzed is guaranteed confidentiality. Throughout the project, only the principal researcher and the research group will have access to the material. After completion of the project all collected material will be archived according to the prevailing rules at Karolinska Institutet.

2:8 Describe previous experience (own and/or others') of the procedure, technique or treatment used: The primarily responsible researcher of the present research has since 2005 worked at MIC Lab with similar Swedish projects based on data from recorded and coded sessions. All the methods used are scientifically proven and established in the scientific community. No risks of complications for any of the methods proposed in this project have been identified through previous studies or in the existing literature.

3. Information about the research participants

3:1 How are research participants chosen? The five Swedish county councils (i.e., Skåne, Stockholm, Sörmland, Värmland and Västernorrland) involved in the study have all responded positively to an initiative funded by the National Board of Health and Welfare to evaluate the MI-training programs in the Swedish county councils. All the 20 Swedish county councils were approached, but the other 16 declined to have their programs evaluated. Throughout the study's recruitment period, each training provider from the five involved county councils will serve the research group with lists of workshop participants. All practitioners who participate in the MI trainings in the involved county councils during the period of January first 2013 to June 31 2014 will be invited to the study by phone and email.

3:2 State the relationship between the researchers/leader of the research and those participating in the research: Evaluator

3:3 State the statistical foundation with respect to the size of the population(s) and/or material(s) studied: The primary outcomes for this trial is the summary values for each of the groups on each of the seven MITI proficiency measures (Empathy, MI spirit, Adherent behaviors, Non-adherent behaviors, Reflection to question ratio, Percent open questions, and Percent complex reflections) at pre-training, post-training, and at the six-month follow-up assessment. The power analysis shows that we need to include 200 participants to attain a power of at least .80 with $p<.05$, and a between-group effect size of .40.

3:4 State if participants in the research may be included in several studies, either simultaneously or in another study or other studies closely linked to this one. If so, what kind of research? The participants will not be included in any other studies.

3:5 What insurance cover is there for research participants taking part in the project? The participants are insured within their workplaces.

3:6 What financial remuneration or other benefits are participants in the research entitled to and when is this to be paid? Participation is voluntary and there is no cost or compensation for participating in the study. Participants randomized to the RWT+TS group will receive six individual monthly sessions of telephone supervision.

4. Information and consent

4:1 The procedure involved and the content of the *information* that is given when subjects are asked to participate in the research:
All the practitioners invited to the study will be contacted by telephone by the research team. The practitioners will be informed about the study's purpose and procedures, the type of data collected and how the data will be processed. The practitioners will also be informed that participation is voluntary and that all participants can withdraw at any point. All the practitioners will then get an email containing the same information and a consent form (Appendix 4, 4b).

4:2 How is *consent* to be obtained and from whom? All the practitioners invited to the study will get an email with information about the study's purpose and procedures, the type of data collected and how the data will be processed. The email will also contain information regarding the voluntary participation, and that all participants have the right to choose to withdraw at any point. The practitioners who choose to participate will answer the email stating informed consent. The email will be printed and kept locked in a filing cabinet at Karolinska Institutet.

5. Considerations in the light of research ethics

5:1 Describe the risks that participation might entail: The risk of privacy violation and/or discomfort is judged as minor as is the risk of complications. The research team is subject to strict confidentiality. All names and personal data will be replaced with a code number and no single participant can be identified in the final material.
The recordings will be renamed with the same participant's code number and all MIC Lab web connections are encrypted. Throughout the project, only the principal researcher and the research group will have access to the identifying material. The actors role-playing standardized patients have documented skills and will not present a risk of discomfort for the participants. The coders who will perform the monthly telephone supervision sessions will be responsible for not putting the participants in a vulnerable situation.

5:2 Describe the foreseeable benefits for the participants in the project: The participants who choose to participate have a vested interest to evaluate and improve their clinical practice of MI. This study will provide them with a detailed feedback of clinical practice, which can be perceived as a benefit. In addition, this study provides knowledge about the most effective form of training to achieve and maintain proficiency in MI. Improved MI education can imply a long-term gain for practitioners in the form of long-term improved educational services.

5:3 Evaluate the relationship between risk and benefit for participants: The potential risks of discomfort or fear of privacy violation are deemed to be small compared to the benefits in terms of the detailed feedback of clinical practice the participants will receive. However, there is always a risk in this type of project involving human subjects. This must be weighed against benefits in terms of improved educational services. Overall, the benefits outweigh the risks.

5:4 In a broader perspective, identify and specify which ethical problems, such as risk versus benefit, can arise as a part of or as a result of the project: There is no expected risk in participating in this study. However, the study has potential benefits for both the participants and their clients, as well as for society at large. The purpose of using evidence-based-practice in community-based settings is to take the latest evidence from research and guide patient care to achieve the best possible outcomes for patients. The integration of evidence-based practice into community-based settings has therefore a lot of benefits for health care since it can lead to improved patient outcomes. Training programs for practitioners is an important variable in the process of adopting evidence-based-practice into routine care. Overall, the benefits of this study outweigh the risks.

6. Presenting the results

6:1 How are both the entity principally responsible for the research and research collaborators guaranteed access to data (to be stated when the research is commissioned) and who is responsible for processing data and writing reports? The Swedish National Board of Health and Welfare fund this study. The researcher primarily responsible for conducting the project, Lars Forsberg, will be responsible for data collection, data processing and report writing. The research group will submit a report to the Swedish National Board of Health and Welfare in September 2014.

6:2 How will the results be made publicly available? Will the study be sent for publishing in a journal or published in some other manner?
Results will be made public via the publication of reports and in scientific journals. Results will also be presented at national and international congresses.

6:3 In what manners will the right to integrity of those participating in the research be guaranteed when the material is made public or is published? All results from this study will be reported on group level. Reporting is completely anonymous and participating individuals cannot otherwise be identified.

7. Reporting the financial circumstances and dependencies

7:1 When the research is commissioned: The Swedish National Board of Health and Welfare, Avdelningen för kunskapsstyrning, Nationella riktlinjer, 106 30 Stockholm, SWEDEN. Anna Månsdotter, + 46 (752) 473 498.

7:2 Give an account of any financial agreements with a responsible body or any other financiers (name, amount): SEK 2 175 109 (Appendix 12).

7:3 Give an account of the interests of the responsible research body, the principal researcher and of participating researchers: N/A

8. Signatories
Please contact the corresponding author Maria Beckman (maria.beckman@ki.se) for the original Swedish document including signatories.

9. List of appendix
Please contact the corresponding author Maria Beckman (maria.beckman@ki.se) for the original Swedish document including the appendix.

Various parts of the original text have been slightly condensed or expanded to increase the readability for English readers.
